# Discovery of urinary metabolite biomarkers of psychiatric disorders using two-sample Mendelian randomization

**DOI:** 10.1186/s12888-026-08133-7

**Published:** 2026-05-07

**Authors:** Jihan K. Zaki, Jakub Tomasik, Jade A. McCune, Oren A. Scherman, Sabine Bahn

**Affiliations:** 1 Department of Chemistry, University of Cambridge, Lensfield Rd, Cambridge, CB2 1EW UK; 2 Department of Chemical Engineering and Biotechnology, University of Cambridge, Philippa Fawcett Drive, Cambridge, CB3 0AS UK

**Keywords:** Psychiatric disorders, Mendelian randomization, Biomarkers, Metabolites, GWAS

## Abstract

**Background:**

Mental health disorders cause substantial patient suffering, which could be alleviated through early diagnostic biomarkers. While biomarker discovery is costly, genetic methods utilizing data from large-scale studies, such as Mendelian randomization, may provide a cost-effective approach.

**Methods:**

A two-sample Mendelian randomization analysis was conducted to identify potential urinary biomarkers of seven psychiatric disorders using summary statistics from GWAS data.

**Results:**

The analysis revealed 67 analyte-disorder associations, of which 21 were exclusive to a single disorder. Notable associations were observed between tyrosine and schizophrenia (β = -0.041, SE = 0.013, Q = 0.027), creatine and bipolar disorder (β = -0.077, SE = 0.019, Q = 0.002), pyridoxal (β = 0.10, SE = 0.03, Q = 0.042) and ferulic acid 4-sulfate (β =  0.077, SE = 0.025, Q = 0.037) to anorexia nervosa, and N, N-dimethylglycine to ADHD (β = -0.39, SE = 0.11, Q = 0.008).

**Conclusion:**

The results identify candidate urinary biomarkers and demonstrate the utility of genetic instruments for biomarker discovery, warranting experimental validation in independent cohorts.

**Clinical trial number:**

Not applicable.

**Supplementary information:**

The online version contains supplementary material available at 10.1186/s12888-026-08133-7.

## Background

Psychiatric disorders pose significant diagnostic challenges, leading to substantial personal and economic burden worldwide. The 12 most debilitating psychiatric disorders are estimated to annually cause 125 million disability adjusted life years globally [1], and economically the direct and indirect costs of psychiatric disorders in the UK alone are estimated to account for 4.5% of the gross domestic product, or £70B-£100B every year [2]. Only the three most debilitating psychiatric conditions, i.e., major depressive disorder, schizophrenia, and bipolar disorder, have each been estimated to incur annual societal costs of £5B-£8B in England alone [3–5].

Although early intervention and treatment have been associated with improved long-term outcomes [6–8], delays in the diagnosis of psychiatric disorders are common. The difficulty in diagnosing psychiatric diseases lies within the current frameworks, which are primarily interview-based methods [9, 10], as well as the lack of biological consistency within the disorders [11]. As a result, 39% of patients with a severe psychiatric disorder are initially misdiagnosed [12]. It has been reported that the average diagnostic delay for bipolar disorder is 6.5 years [13], and the mean duration of untreated psychosis has been estimated to be up to 2.9 years [14]. Early and accurate diagnosis and intervention could significantly mitigate the severe burden and costs associated with mental health disorders. This could be facilitated by the discovery of consistent and actionable biomarkers in easily accessible patient samples, which could additionally provide a valuable means to improve biological understanding of psychiatric diseases.

Among other biological patient samples, urine holds great promise as a potential source of biomarkers. However, it is commonly overlooked by the scientific community for nearly all disorders. Publications listed in PubMed for blood-based biomarker research outnumber urinary biomarker publications ten-fold. Despite being often overlooked, urine mostly retains the biomarker signal observed in plasma, as a substantial proportion of urinary metabolite concentrations are filtered directly from plasma [15]. Furthermore, urine has significant advantages as a biomarker source compared to others due to the ease and non-invasive nature of sample collection, and the abundance of sample volume. Discovery of urinary markers could introduce an opportunity for a simple, non-invasive, and longitudinal biomarker assessment as well as point-of-care testing for high risk of disease or relapse.

The high clinical potential of urinary biomarkers can be fully utilised with careful study design and appropriate methodology. Computational methods could overcome the challenges of urinary biomarker discovery in psychiatric diseases, including the variation in analyte concentrations [16, 17] as well as the significant impact of potential confounding effects. One such method is a genetic analysis called Mendelian randomization (MR) [18], which utilizes the principles of instrumental variable analysis and applies them to genetic data to evaluate the effects of a given exposure (i.e., an analyte) on a selected outcome (i.e., risk of disease) when the effect of the genetic variability on both are known.

We aimed to discover genetically linked urinary markers for seven psychiatric disorders potentially capable of differential diagnosis. A two-sample MR study was conducted using exposure data obtained from a meta-analysis of urinary metabolite GWAS studies and urinary metabolite associations from the GWAS catalog, as well as outcome data obtained from the most up-to-date psychiatric disorder GWAS data. Additionally, sensitivity analyses were conducted, including tests for horizontal pleiotropy, heterogeneity, confounder associations, as well as the determination of potential mechanistic reasons for altered expressions, to validate MR assumptions and ensure the robustness of the identified biomarker candidates.

## Methods

### Compilation of the exposure data

The exposure association data were obtained from a meta-analysis of urinary metabolite GWAS described in detail by Zaki et al. [19], as well as results from the GWAS catalog under the trait *urinary metabolite measurement* (EFO_0005116). In brief, the meta-analysis combined results from five urinary metabolite GWAS [20–24] using a sample size-based meta-analysis method [25], identifying 2248 significant associations for 14 analytes (*P* < 7.1 × 10^−9^) prior to clumping. The identified SNPs from the meta-analysis were combined with 195 associations for 152 analytes obtained from the GWAS catalog [20, 21, 23, 24, 26–30], excluding SNPs duplicated in the catalog as well as mislabelled studies conducted in other sample types. All studies contained European ancestry populations; however, some studies differed in confounder profiles such as obesity and age. Exclusively genome-wide significant associations (*P* < 5 × 10^−8^) were selected for the analysis from the GWAS catalog. An F-statistic was calculated for all exposure associations to evaluate weak instrument bias [18]. For associations obtained from the GWAS Catalog, only instruments exceeding the conventional threshold of F > 10 were included. For SNP-analyte associations derived from the meta-analysis, F-statistics for each individual study are reported in Supplementary Table 1.

### Psychiatric disorder GWAS data collection

The most recent summary statistics GWAS data for each assessed psychiatric disorder were selected from meta-analyses conducted by the Psychiatrics Genomics Consortium (PGC), which were primarily based on European ancestry populations and did not have patient overlap with the exposure datasets. The seven psychiatric disorders selected for this study included attention deficit hyperactivity disorder (n_ADHD_ = 20183, n_CTRL_ = 35191) [31], anorexia nervosa (n_ANO_ = 16996, n_CTRL_ = 55525) [32], autism spectrum condition (n_ASC_ = 18381, n_CTRL_ = 27969) [33], bipolar disorder (n_BD_ = 41917, n_CTRL_ = 371549) [34], major depressive disorder (n_MDD_ = 61991, n_CTRL_ = 302043) [35], schizophrenia (n_SCZ_ = 74776, n_CTRL_ = 101023) [36], and Tourette’s syndrome (n_TS_ = 4819, n_CTRL_ = 9488) [37]. For psychiatric GWAS studies where samples may have partially overlapped with the exposure GWAS studies, cohorts contributing to both datasets were excluded from the exposure dataset, and consequently the final results, to prevent bias. Further details regarding both exposure and outcome cohorts are presented in Supplementary Tables 2–9.

### Mendelian randomization

Two-sample MR was conducted to genetically assess putative causal associations of analytes selected in the meta-analysis and the GWAS catalog to the seven psychiatric disorders. MR analysis was carried out using the TwoSampleMR [38] package in R version 4.2.0. For the significant analyte-SNP associations from the meta-analysis, MR was performed separately between each of the meta-analysed studies and the outcome datasets, with MR estimates subsequently combined using the inverse variance weighted (IVW) meta-analysis method [25]. This two-step process was necessary due to inconsistent measurement methods, scales, and log transformations used for individual analytes included in the meta-analysis, which caused their beta coefficients and standard errors to be incomparable and prevented them from being combined into a single coefficient. For the exposure data from the GWAS catalog, standard MR was performed.

For each of the analyses, closely located (1000 kb) and correlated (r^2^ > 0.001) SNPs in the exposure dataset were combined by clumping using the European population-based reference panel from the 1000 Genomes Project [39]. For exposure SNPs not found in the outcome dataset, highly correlated (r^2^ > 0.8) linkage disequilibrium (LD) proxy replacements in the outcome dataset were identified using the European population from the 1000 Genomes Project as a reference, and the LDlinkR [40] package in R. The IVW ratio MR method was used for metabolites with at least two SNP associations, and the Wald ratio (WR) MR method was used for metabolites with only a single SNP association [41]. For metabolites with three or more SNP associations, sensitivity analyses were conducted using the weighted median, weighted mode, and MR-Egger methods to assess consistent directionality and effect sizes [42]. Cochran’s Q test was applied for metabolites assessed using the inverse variance weighted method to assess heterogeneity between effect size estimates [42]. The Benjamini-Hochberg false discovery rate (FDR) method was used to correct for multiple comparisons across all analyte-disorder pairs tested, with the significance threshold set to Q < 0.05. The FDR approach was selected over more conservative methods such as the Bonferroni correction because the tested metabolites and psychiatric disorders are likely correlated through shared genetic architecture, for which the Bonferroni correction may be overly conservative. The Q < 0.05 threshold controls the expected proportion of false discoveries among significant results to 5%.

### Assumption validation

The three assumptions of MR analysis state that: (1) the genetic instrument must be associated with the exposure, (2) the genetic instrument must not directly influence the outcome (horizontal pleiotropy), and (3) the genetic instrument must not be associated with potential confounders of the exposure and the outcome [18]. These assumptions were assessed for all results which passed the significance threshold following multiple comparison correction. The first was assessed through the significance level of the exposure associations (*P* < 5 × 10^−8^) in either the GWAS catalog or the meta-analysis. To assess the second and third assumptions, SNP-trait associations were examined using the PhenoScanner database [43, 44], with genome-wide significant associations to either the disorder or known demographical confounders invalidating the assumptions. Additionally, MR-Egger intercept was used to assess metabolites with more than three SNP associations, with significant associations indicating horizontal pleiotropy [42].

## Results

### Mendelian randomization

In the present work, the genomic causal effect of 163 urinary analytes on seven selected psychiatric disorders was assessed using two-sample MR, with a total of 4852 analyte-SNP associations combined from the meta-analysed studies (4657) and the GWAS catalog (195) in the exposure dataset, and 54,746,959 unique SNP measurements in the outcome dataset. Prior to assumption validation, 68 statistically significant metabolite-disorder associations were discovered between 44 unique analytes and the mental health conditions ADHD, anorexia, BD, and schizophrenia following multiple comparison adjustment.

### Assumption validation and sensitivity analyses

To ensure that the first assumption of MR is met, and the instrumental variables are robustly associated with exposure, the analysis included only those SNP-analyte associations that were genome-wide significant (*P* < 5 × 10^−8^) in either the GWAS catalog or the meta-analysis. The second and third assumption of MR was assessed using SNP-trait associations from the PhenoScanner database. The analysis revealed a genome-wide significant association of alcohol intake frequency with rs2287921 and rs281408, which were both significantly associated with fucose. Therefore, the association of fucose to schizophrenia (β = 0.09, SE = 0.017, Q = 3.65 × 10^−5^) was excluded from the list of results. No other analysed SNPs were found to violate the assumptions of MR. Horizontal pleiotropy was not found to be present in any of the SNP-trait associations. Further sensitivity analyses showed that for associations whose exposure data were obtained from multiple studies, the MR estimates were consistent between individual source studies, as shown in Supplementary Figs. 1 and 2. Additionally, no heterogenic effects were observed for SNPs involved in any of the significant associations (Supplementary Table 10). For associations whose exposure data included three or more SNP instruments, sensitivity analyses using the weighted median, weighted mode, and MR-Egger methods showed consistent effect sizes and directionality (Supplementary Fig. 3). MR-Egger intercept tests did not indicate the presence of horizontal pleiotropy in any of the assessed associations (Supplementary Table 11). A sensitivity analysis including overlapping exposure and outcome datasets showed that for BD and anorexia, overlap with the UK Biobank exposure cohort affected four analytes (creatinine, microalbumin, potassium, and sodium), none of which were among the significant associations. For MDD, overlap existed through the SHIP, SHIP-Trend, and UK Biobank cohorts; however, no significant associations were identified for MDD. No overlap was present for the schizophrenia, TS, ASC or ADHD cohorts. In all cases, exposure SNPs originating from overlapping cohorts were excluded from the corresponding analyses.

### Common markers of psychiatric disorders

**Fig. 1:**
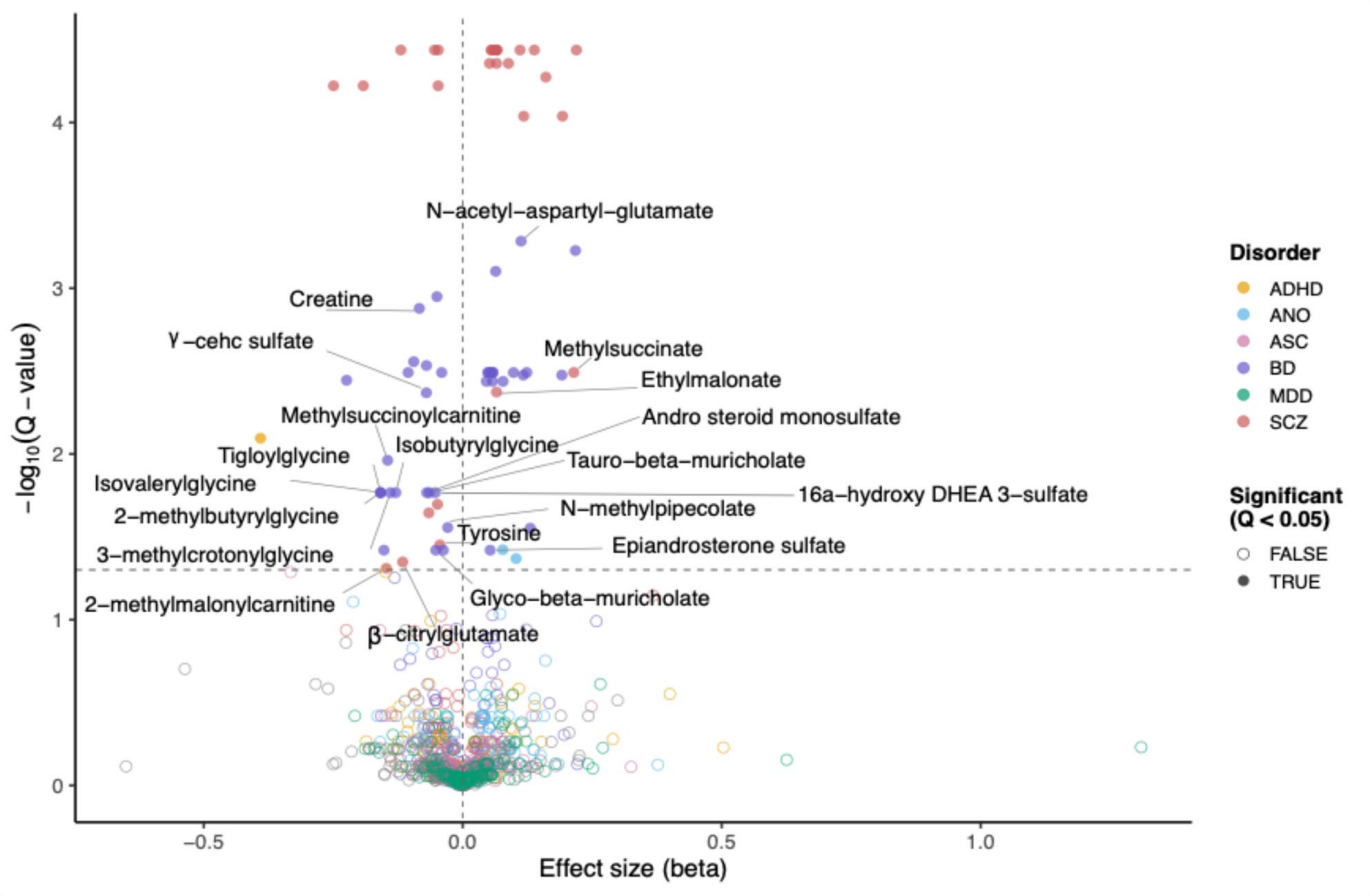
Volcano plot of urinary metabolite-disorder associations assessed by two-sample Mendelian randomization. Effect size estimates (beta) are shown on the X-axis and − log₁₀ Q-values are shown on the Y-axis. Points are colour-coded by psychiatric disorder, with filled points indicating statistically significant associations and open points indicating non-significant associations. The horizontal dashed line represents the significance threshold (Q = 0.05). Labelled metabolites represent associations specific to a single disorder

Following the assumption validation, 67 metabolite-disorder associations remained significant between 43 unique analytes and the respective disorders, with no significant associations found for ASC, MDD, or TS. All significant metabolite-disorder associations following meta-analysis and assumption validation are presented in a volcano plot in Fig. 1, as well as Supplementary Table 12.

**Fig. 2:**
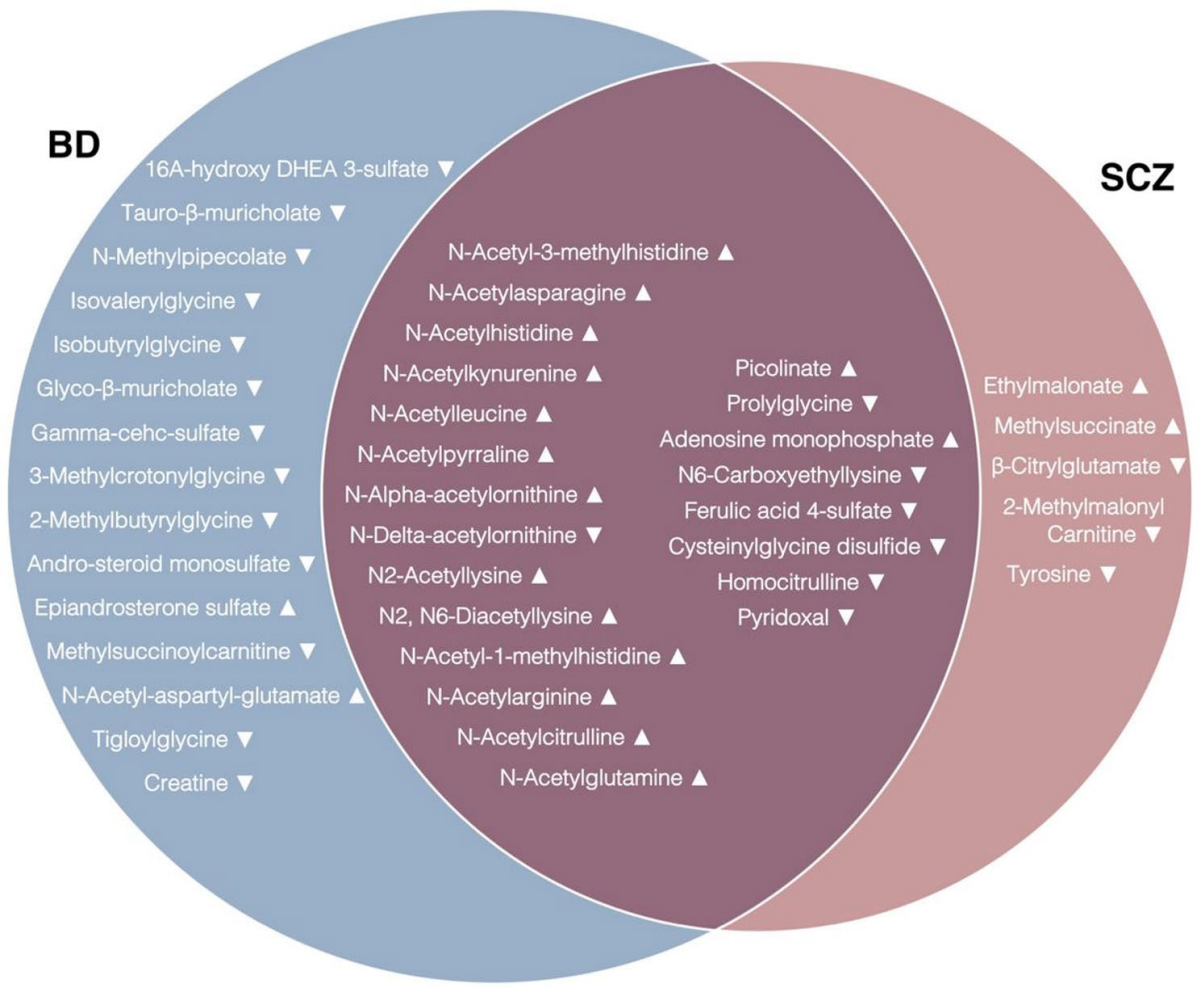
Venn diagram of significant analyte association for schizophrenia (SCZ), and bipolar disorder (BD). Effect size directionality has been indicated with triangles (▲ upregulated, ▼ downregulated). For metabolites in the overlapping region, effect direction was consistent between BD and SCZ for all shared analytes

The largest overlap was observed between biomarker associations to BD and schizophrenia, which overlapped in 22 unique analytes with similar effect sizes and directionality. The extent of overlap between BD and schizophrenia is shown in Fig. 2. The most significant associations overlapping between BD and schizophrenia involved most notably numerous N-acetylated compounds. In addition, anorexia was associated with upregulated pyridoxal (β = 0.10, SE = 0.03, Q = 0.042) and ferulic acid 4-sulfate (β = 0.077, SE = 0.025, Q = 0.037), which also overlapped with BD and schizophrenia, however with opposite directionality.

### Differential markers of psychiatric disorders

**Fig. 3:**
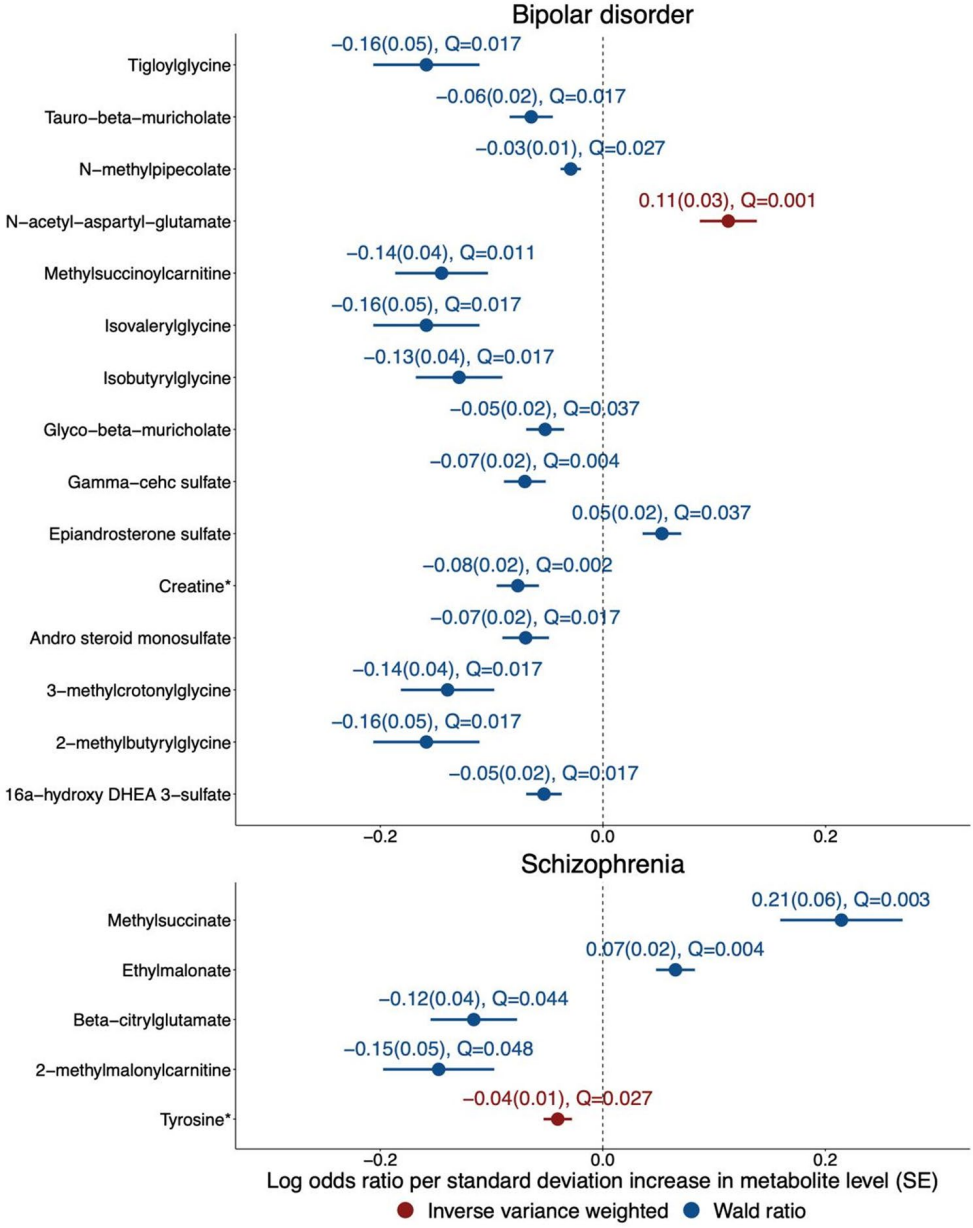
Estimated genomically predicted causal effects of urinary markers on the risk of bipolar disorder and schizophrenia. Urinary markers are shown on the Y-axis and log-odds ratio of bipolar disorder and schizophrenia are shown on the X-axis. Dots represent the mean effect size, with horizontal lines representing standard errors. Combined results from multiple studies are marked with an asterisk (*). Q-values represent P-values adjusted for multiple comparisons

Of the 67 significant metabolite-disorder associations, 21 were exclusive to a single disorder. Of these, five were associated with schizophrenia, 15 with BD, and one with ADHD. The most robust associations were observed between tyrosine and schizophrenia (β = -0.041, SE = 0.013, Q = 0.027), and creatine and BD (β = -0.077, SE = 0.019, Q = 0.002), due to their associations being significant after assessment in multiple studies. The single analyte association to ADHD was N, N-dimethylglycine (β = -0.39, SE = 0.11, Q = 0.008). All non-overlapping analyte associations specific to schizophrenia or BD are shown in Fig. 3.

## Discussion

The present study aimed to discover putative urinary metabolite biomarkers of psychiatric diseases using genetic instruments. To this end, a two-sample MR analysis was conducted using GWAS exposure data obtained from a meta-analysis of urinary metabolite GWAS studies as well as the publicly available GWAS catalog, and the outcome data obtained from the PGC. Genomically predicted causal effects were identified for 67 analyte-disorder associations, of which 21 were specific to a single disease. Most notable findings of the analysis include the common association of N-acetylated compounds to BD and schizophrenia, tyrosine to schizophrenia, creatine to BD, N,N-dimethylglycine to ADHD, as well as pyridoxal and ferulic acid 4-sulfate to anorexia. The overlapping markers, as well as markers of ADHD and anorexia are discussed in the supplementary material. Previously published direct and indirect associations were found in the existing literature for all of the shared putative biomarkers of the disorders, and are discussed below for the non-overlapping biomarkers identified in the present study. Markers overlapping between diseases, as well as the marker of ADHD, are discussed in the supplementary extended discussion in the supplementary material. Furthermore, tyrosine and creatine have been reported to show strong and robust pathophysiological associations with schizophrenia and BD, respectively. Owing to their specificity and the multiple lines of supporting evidence, these biomarkers have potential to differentiate not only patients from healthy controls, but also different diseases from each other.

The 15 significant urinary biomarkers associated uniquely with BD can be grouped into glycine-related compounds (creatine, tigloyl glycine, isovaleryl glycine, isobutyryl glycine, 3-methyl crotonyl glycine, and 2-methylbutyrylglycine), sulfates (gamma-cehc sulfate, epiandrosterone sulfate, 16a-hydroxy DHEA 3-sulfate, andro-steroid monosulfate), cholates (tauro-beta-muricholate, glyco-beta-muricholate), as well as individual compounds, N-acetyl-aspartyl-glutamate, N-methylpipecolate, and methyl succinyl carnitine. Although sulfates, cholates, and the individual compounds have no known associations to BD, existing literature shows that glycine- and glutamate-related compounds have well-established links to the disease.

Glycine and glutamate alterations in plasma have been observed in BD patients during manic phases, irrespective of mood stabilizer treatment [45]. Glutamate and glycine are co-agonists at the N-methyl-D-aspartate receptor (NMDAR), the abnormalities of which have been hypothesized to be associated with symptoms of BD [46]. The specific details of the mechanism of action regarding NMDARs and BD are not yet thoroughly understood, however the results of the MR analysis further support the connection and suggest a pathophysiological role for N-acetyl-aspartyl-glutamate. Additionally, the MR analysis results imply that creatine is inversely correlated with BD. Creatine is a compound commonly linked with energy metabolism in muscle and brain tissue, and can be supplemented dietarily, however in the body it is also synthesized using glycine and other amino acids [47]. Creatine has been extensively linked with BD in past studies because of its relation to brain energy metabolism. Reductions of creatine levels have been identified in several regions of the brain in BD [48, 49], and total creatinine has been shown to be inversely correlated with depressive symptom severity in patients with BD [50]. In a clinical trial of creatine add-on supplementation, the efficacy of creatine in treating depressive symptoms was not statistically significant, however it was shown to significantly improve remission rates in BD patients [51]. Therefore, it can be hypothesized that naturally increased creatine levels could have protective effects in relation to symptoms of BD.

The five urinary analytes with exclusive associations to schizophrenia were tyrosine, methylsuccinate, ethylmalonate, beta-citrylglutamate, and 2-methylmalonylcarnitine. The analytes can be grouped into two categories. Tyrosine and beta-citrylglutamate are metabolically associated with dopamine and glutamate, which are central to the leading hypotheses of the mechanism of schizophrenia. In turn, 2-methylmalonylcarnitine, methylsuccinate, and ethylmalonate are associated with metabolic disturbances in mitochondria, which similarly have been hypothesised to cause schizophrenia.

Methyl succinic acid and ethylmalonic acid are conjugate acids of methylsuccinate and ethylmalonate, respectively. When upregulated in urine, they are biomarkers of the severe neurometabolic condition ethylmalonic encephalopathy (EE) [52], which is characterized by disturbances in the brain, cardiovascular, and digestive systems. EE is caused by disturbances in mitochondrial function through mutations affecting the production of an enzyme which breaks down sulfides in mitochondria. As the MR results imply that upregulated urinary methylsuccinate and ethylmalonate increase susceptibility to schizophrenia, it can be hypothesized that a similar mechanism to that of EE involving mitochondrial dysfunction may exist in schizophrenia. In addition, ethylmalonate in cerebrospinal fluid has been directly associated with schizophrenia in the past [53], suggesting that a mechanistic association may exist between ethylmalonate and the disease. In turn, 2-methylmalonylcarnitine is a short-chain acylcarnitine which has roles in acyl-group compound transport into mitochondria for energy production [54], and it has been linked with ASC through effects on mitochondrial function [55]. Mitochondrial disturbances have been strongly linked with increased risk of schizophrenia [56, 57], however the exact pathomechanism remains elusive. The results of the MR analysis linking ethylmalonate, methylsuccinate, and 2-methylmalonylcarnitine to schizophrenia therefore indicate a possible causal linking factor through effects on mitochondrial function and should be further validated in future studies.

The MR results regarding tyrosine and beta-citrylglutamate are in line with the dopamine and glutamate hypotheses of schizophrenia [58, 59]. Briefly, the dopamine hypothesis states that the psychotic symptoms of schizophrenia stem from hyperactive dopamine neurotransmission, as well as oversensitivity of dopamine receptors [59]. Most current antipsychotic treatments target this system through dopamine receptor blockage [59]. Tyrosine is a precursor amino acid of dopamine and is thus central to the dopaminergic pathway, acting as a limiting factor in the production of dopamine [60]. Observed genomically predicted causal effects from the MR imply that higher levels of tyrosine result in a lower likelihood of schizophrenia development. The results can be interpreted within the context of the dopamine hypothesis as being a result of potentially enhanced conversion of tyrosine into dopamine, however other possibilities also exist. Previous clinical trials have not supported the possibility of a protective effect of tyrosine on schizophrenia symptoms [61], therefore it is more likely that the observed effect is an outcome of a mechanistic step in the symptom development pathway. Other studies have suggested that limited tyrosine transport to the brain through the blood brain barrier could be causal to the alterations of dopamine levels in the brains of schizophrenia patients [62, 63]. Therefore, increased peripheral levels of tyrosine could imply that an excess of tyrosine may mitigate the lower tyrosine transport into the brain, and thus reduce schizophrenia symptoms.

The literature regarding beta-citrylglutamate is limited, however it is a known derivative of glutamate, and concentrations of beta-citrylglutamate decrease with age [64]. The glutamate hypothesis of schizophrenia is similar to the observations of glutamate disruptions in BD, where the downregulated glutamate signalling function of NMDARs are hypothesized to be associated with the cognitive and negative symptoms of schizophrenia [58]. The MR results imply that decreased urinary beta-citrylglutamate increases the risk of developing schizophrenia, which may be due to lower baseline levels of glutamate resulting in decreased glutamate signalling. Taken together, the present results are in line with the dopamine and glutamate hypotheses of schizophrenia and indicate urinary tyrosine and glutamate as potential biomarkers.

The present MR analysis uniquely contributes to the pathophysiological understanding of the assessed disorders by providing genetic evidence supporting the causal direction of previously observed metabolite-disorder associations. Unlike observational studies, which cannot distinguish whether metabolite alterations are a cause or consequence of disease, the MR framework leverages genetic variants as instruments to infer the direction of effect. For instance, the observed inverse association of tyrosine with schizophrenia supports the hypothesis that reduced peripheral tyrosine availability may contribute to dopaminergic dysfunction, rather than being solely a downstream consequence of disease processes or treatment effects.

The observed urinary analyte-disorder associations, if validated, have the potential to be used in a diagnostic biomarker panel. This is due to the sheer number of markers exclusive to a single disease, specifically for BD and schizophrenia. The collective predictive value of the identified biomarkers could surpass the threshold for utility in a clinical test. A significant benefit of the discovered biomarkers concerns the target disorders. BD and schizophrenia are commonly misdiagnosed with each other, with an estimated 24% of schizophrenia patients being misdiagnosed with BD [12, 65], and one-third of BD patients receiving an initial diagnosis of schizophrenia [66]. Therefore, a diagnostic panel would only need to be able to accurately differentiate the two disorders from each other to be clinically relevant. Additionally, for high-risk individuals such as relatives of patients, a point-of-care diagnostic assessment could be developed to identify early indicators of either disorder, owing to the non-invasive nature of urinary measurements. Notably, only the causal association of tyrosine with schizophrenia can be considered particularly robust among the findings, as it was identified across multiple independent studies and supported by consistent sensitivity analyses. For the remaining associations estimated using the Wald ratio method with single SNP instruments, causal interpretation should be made with caution, as the absence of multiple instruments precludes comprehensive sensitivity analyses for horizontal pleiotropy.

The present study highlights the robust capability of computational methods to pre-emptively identify high-priority targets in biomarker discovery in addition to traditional methods. The current landscape in biomarker discovery does not utilize computational resources to its full extent. While MR studies have been carried out in the past for biomarkers of mental health disorders [67, 68], the intent is often to exclusively identify genomically predicted causal effects and not disease biomarkers. Additionally, in the vast majority of biomarker discovery trials, computational screening is not utilized for target identification. As the library of GWAS studies expands, the use of computational biological assessment through MR should be more accessible and applicable to a wide variety of disorders and markers. Therefore, the use of computational methods for biomarker candidate screening should be broadly implemented to maximize the probability of success in clinical trials, especially considering the costs of conducting such studies, and the low cost of MR analysis. Furthermore, incorporating polygenic risk score (PRS) analyses could complement the MR approach by examining whether individuals with high genetic risk exhibit noticeable deviations in urinary biomarkers. This could provide deeper insight into the metabolite-disease associations at individual patient level.

The present study is subject to a number of limitations. First, urinary analyte levels are significantly impacted both between and within individuals and depend on changes in daily activities such as diet, hydration, or activity levels [16, 17]. When considering that the majority of the identified markers show small effect sizes, the observation and validation of the markers in real samples could be challenging. Second, there is a lack of availability of comprehensive urinary GWAS data. Urinary analyte GWAS compared to other GWAS studies typically have small sample sizes, measure a limited number of analytes, and use more variable scales, which limits the number of SNPs available for analysis and necessitates the use of non-standard MR methods, such as the one used in the present study. Additionally, palindromicity of the analysed SNPs was not evaluated in the analysis due to a lack of allele frequency data in the majority of the exposure datasets. Third, the reliance on single SNP instruments for many metabolites necessitated the use of the Wald ratio method. However, the use of single SNP instruments can limit the robustness of causal estimates and precludes the use of sensitivity analyses that require multiple instruments, such as the MR-Pleiotropy RESidual Sum and Outlier (PRESSO) method. To ensure robustness of the analysis and minimize weak instrument bias, we opted for a stringent P-value threshold (*P* < 5 × 10^–8^) when selecting exposure and outcome SNPs. However, this limited the number of available SNPs, potentially resulting in false negative findings. Fourth, in the outcome datasets, the SNP associations are assessed against healthy controls and not against other disorder groups, therefore it is feasible that some differential biomarkers identified in this study may overlap in real patient samples. Fifth, the assessed GWAS studies in the analysis are primarily based on European populations, therefore the analysis might not translate to other ethnic populations. Furthermore, there is potential bias introduced by ancestry differences between some of the exposure and outcome GWAS datasets. While our exposure data were exclusively derived from European ancestry populations, the schizophrenia, bipolar disorder, and ADHD cohorts included a small minority of samples from other ancestry groups. As such, the results may not apply to other ethnic groups. Furthermore, the GWAS summary statistics used for both exposures and outcomes are derived from observational cohorts, which may be subject to residual confounding or selection biases that the MR framework cannot fully eliminate. Additionally, some of the psychiatric disorder GWAS datasets used in this study may not represent the most recent or largest available datasets. The use of smaller outcome GWAS datasets may have reduced the statistical power of the analysis, potentially contributing to the absence of significant associations for MDD, ASC, and TS. Sixth, a small number of SNP-analyte associations from the meta-analysis exhibited F-statistics below the conventional threshold of F > 10 in individual contributing studies, specifically for alanine, 2-hydroxybutyrate/2-hydroxyisobutyrate, and trimethylamine. However, as weak instrument bias in two-sample MR shifts effect estimates towards the null, this would reduce statistical power rather than inflate false positive rates. Furthermore, the affected analytes did not yield significant associations in any of the individual contributing studies, including those with strong instruments (F > 10), suggesting that the null findings for these metabolites are unlikely to be artefacts of weak instrument bias. Lastly, we were unable to perform Multivariable Mendelian Randomization (MVMR), as implementing MVMR requires multiple overlapping independent genetic instruments for each exposure. In our study, most metabolites had only one associated SNPs, making robust MVMR analyses infeasible.

## Conclusion

In conclusion, we identified 21 urinary metabolite candidates with potential relevance to the diagnosis and differentiation of psychiatric disorders using a two-sample MR approach. Of the putative biomarkers, five were associated with schizophrenia, 15 with BD, and one with ADHD. Tyrosine for schizophrenia and creatine for BD were considered the most robust candidates, owing to their effects being identified across multiple studies and supported by consistent sensitivity analyses. The findings indicate that urine could be a valuable source of biomarkers for psychiatric disorders and highlight the utility of computational and genetic analyses for the purposes of biomarker candidate identification. However, the transition from genetically predicted associations to validated clinical biomarkers requires several validation steps, including targeted metabolomics in independent case-control urine samples to confirm the observed associations, replication across multiple independent cohorts to assess generalisability, and evaluation of the combined predictive performance of the identified biomarker panels for differential diagnosis of BD and schizophrenia. With such validation, the identified candidates could contribute to the development of a non-invasive biomarker platform for an improved diagnosis of psychiatric disorders.

## Supplementary Information

Below is the link to the electronic supplementary material.

Supplementary Material 1

Supplementary Material 2

Supplementary Material 3

## Data Availability

The data used in this study are publicly available as follows: summary statistics for the psychiatric disorders were obtained from the Psychiatric Genomics Consortium (PGC) website (https://pgc.unc.edu/for-researchers/download-results/). The specific datasets used are from the studies cited in the manuscript. The GWAS data for the urinary metabolites used as exposures are available as supplementary data. Analysis code is available from the corresponding author upon reasonable request.

## Abbreviations

ADHD: Attention Deficit Hyperactivity Disorder
ANO: Anorexia Nervosa
ASC: Autism Spectrum Condition
BD: Bipolar Disorder
CTRL: Control
EE: Ethylmalonic Encephalopathy
EFO: Experimental Factor Ontology
F: F-statistic
FDR: False Discovery Rate
GWAS: Genome-Wide Association Study/Studies
IV: Instrumental Variable
IVW: Inverse Variance Weighted
LD: Linkage Disequilibrium
MDD: Major Depressive Disorder
MR: Mendelian Randomization
MVMR: Multivariable Mendelian Randomization
NMDAR: N-Methyl-D-Aspartate Receptor
PGC: Psychiatric Genomics Consortium
PRESSO: Pleiotropy RESidual Sum and Outlier
PRS: Polygenic Risk Score
Q: Q-value (Benjamini–Hochberg FDR-adjusted P-value)
SCZ: Schizophrenia
SE: Standard Error
SHIP: Study of Health in Pomerania
SNP: Single Nucleotide Polymorphism
STROBE-MR: Strengthening the Reporting of Observational Studies in Epidemiology – Mendelian Randomization
TS: Tourette’s Syndrome
UK: United Kingdom
WR: Wald Ratio
β: Beta coefficient (effect size estimate)
